# Anatomic modeling using 3D printing: quality assurance and optimization

**DOI:** 10.1186/s41205-017-0014-3

**Published:** 2017-04-26

**Authors:** Shuai Leng, Kiaran McGee, Jonathan Morris, Amy Alexander, Joel Kuhlmann, Thomas Vrieze, Cynthia H. McCollough, Jane Matsumoto

**Affiliations:** 1 Department of Radiology, 200 First Street SW, Mayo Clinic, Rochester, 55901 MN USA; 2 Division of Engineering, 200 First Street SW, Mayo Clinic, Rochester, 55901 MN USA

**Keywords:** Quality assurance, 3D printing, Imaging, Segmentation, Phantom, Computed tomography (CT)

## Abstract

**Background:**

The purpose of this study is to provide a framework for the development of a quality assurance (QA) program for use in medical 3D printing applications. An interdisciplinary QA team was built with expertise from all aspects of 3D printing. A systematic QA approach was established to assess the accuracy and precision of each step during the 3D printing process, including: image data acquisition, segmentation and processing, and 3D printing and cleaning. Validation of printed models was performed by qualitative inspection and quantitative measurement. The latter was achieved by scanning the printed model with a high resolution CT scanner to obtain images of the printed model, which were registered to the original patient images and the distance between them was calculated on a point-by-point basis.

**Results:**

A phantom-based QA process, with two QA phantoms, was also developed. The phantoms went through the same 3D printing process as that of the patient models to generate printed QA models. Physical measurement, fit tests, and image based measurements were performed to compare the printed 3D model to the original QA phantom, with its known size and shape, providing an end-to-end assessment of errors involved in the complete 3D printing process. Measured differences between the printed model and the original QA phantom ranged from -0.32 mm to 0.13 mm for the line pair pattern. For a radial-ulna patient model, the mean distance between the original data set and the scanned printed model was -0.12 mm (ranging from -0.57 to 0.34 mm), with a standard deviation of 0.17 mm.

**Conclusions:**

A comprehensive QA process from image acquisition to completed model has been developed. Such a program is essential to ensure the required accuracy of 3D printed models for medical applications.

## Background

First used in manufacturing, medical applications of 3D printing or additive manufacturing have been rapidly developing. Using a patient’s own medical image data, 3D printing can be used to create individualized, life-size patient-specific models. These models are increasingly being used as aids in surgical planning for complex cases [1–11]. 3D models can contribute to surgical procedures by providing surgeons with an accurate life size physical reproduction of the anatomy of interest. In addition, models offer unique educational opportunities, including simulation for resident training [12, 13]. Research applications include patient-specific imaging and therapeutic phantoms used for advancing imaging techniques, reducing radiation dose, and conducting treatment planning and dose verification in radiation therapy [14–21]. Additional applications include the development of patient specific surgical guides and the reproduction of forensic models.

3D printing offers advantages over conventional manufacturing technologies. Individualized single models can be created as needed in a clinical setting with relatively low cost in a fairly short time frame. Depending on the type of 3D printing technology used, models can be printed with varying material types, colors, and mechanical properties with potential for sterilization. 3D printing is more efficient and less costly than standard manufacturing technologies and is uniquely suited to contribute to individualized patient care.

While the role of 3D printing in medicine is rapidly expanding and will certainly be a part of medical care going into the future, it is important that quality assurance (QA) programs are in place as part of the development and maintenance of a 3D printing program. To develop a QA program, it is important to identify the steps involved in generating a 3D model. There are essentially three steps involved in 3D printing in medicine: (1) Step one involves the acquisition of 3D volumetric images of the patient. (2) Step two is to separate out the anatomy of interest from surrounding structures and output the segmented virtual models as stereolithography (STL) files. This step also includes the editing of the original segmented objects, such as wrapping and smoothing. (3) Step three is to print the physical models and clean them. While the accuracy of medical imaging is a critical first step in 3D printing, the additional elements of segmentation and processing of imaging data and the technical aspects of the 3D printing process can all affect the accuracy of the final 3D printed model. Each of these production steps should be individually evaluated, analyzed and optimized. Medical confidence in the accurate representation of patient anatomy and pathology is a necessary component of medical 3D printing applications.

A successful QA program requires input from all stakeholders involved in the 3D printing process. As such, it is critical to build an interdisciplinary QA team. Typical stakeholders include: 1) Surgeons or physicians who order the model know the clinical need, and are the end users of the model; 2) Interpreting radiologists who possess expertise in acquiring and interpreting the imaging study; 3) Medical physicists who are experts in the imaging technology and ensure exams are performed with optimized scanning and reconstruction techniques; they also have extensive experience and leadership roles for the QA programs in a radiology department; 4) Technologists who perform the imaging and segmentation; 5) Engineers and operators who do variable amounts of segmentation, model design and printer maintenance. An essential component of a successful QA program is effective communication between these team members to ensure creation of a high quality model.

A QA program with validation, verification, and documentation to assure accuracy and quality is therefore a key component of 3D printing. Fortunately, radiology departments have significant expertise in the development of QA programs and this experience can be adapted to medical 3D printing. Expansion of QA programs to include evaluation of the unique characteristics of 3D printing technology and segmentation processes forms the basis of a medical 3D printing QA program. The purpose of this paper is to give an overview of the QA program that has been developed at our institution to assess the accuracy and precision of each step of the 3D printing process.

## Methods

We have developed a systematic approach that involves QA for each of the major steps of 3D printing: imaging, segmentation and processing, and printing. In the following subsections, we will discuss the appropriate QA process for each of these three major steps.

### QA and optimization of image acquisition

The first step of anatomic modeling is to acquire volumetric image data of the patient. The quality of the image data (i.e. spatial resolution, signal-to-noise ratio, artifacts) has a direct impact on the accuracy and efficiency of the following steps, e.g. segmentation, processing, and printing. Therefore, thorough control of the quality of the acquired image data plays a critical role as it directly affects the quality of the final model.

CT and MRI are the most common imaging modalities used to generate 3D volumetric data for the purpose of anatomical modeling. At most institutions, CT and MRI scanners are accredited by an accreditation organization, such as American College of Radiology (ACR). As part of the accreditation, a QA program is required that includes the performance of routine tests for all scanners. Tests are performed on a daily, monthly and annual basis depending on the requirements specified by the accreditation organization. During the QA process, geometric accuracy and spatial resolution are routinely checked [22, 23].

**Fig. 1:**
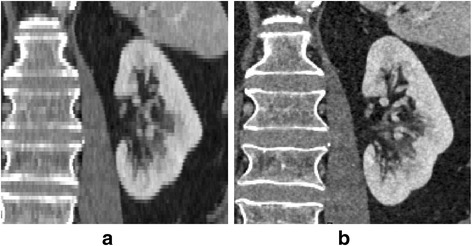
**a** kidney model made off *3 mm slice thickness* showed stairwell artifacts, while they were not visible on the model made off *0.6 mm slice thickness* (**b**)

**Fig. 2:**
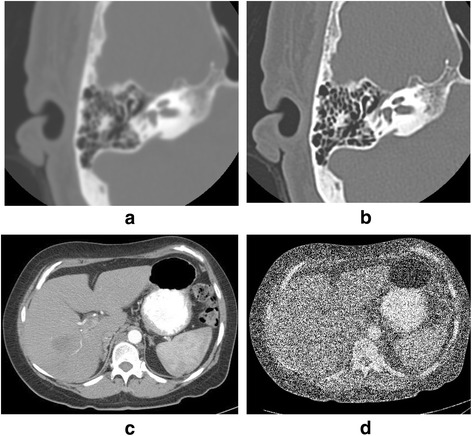
Temporal bone (**a**, **b**) and liver (**c**, **d**) images reconstructed with soft (**a**, **c**) and sharp kernels (**b**, **d**)

As scan and reconstruction parameters directly affect image quality, imaging protocols need to be optimized to meet the needs of 3D modeling. For example, lower tube potential (kV) can be used in CT to increase the enhancement of iodine contrast when building vascular models [24]. Contrast injection and bolus timing can be adjusted to separate arterial and venous phases of the contrast enhancement. Image slice thickness and reconstruction kernel are important factors influencing the spatial resolution and image noise. Thick images can generate discontinuous, stair-step like boundaries on the segmented model (Fig. 1). The reconstruction kernel impacts both spatial resolution and image noise, which need to be balanced based on particular applications. For models with fine structures, such as the temporal bone, a sharp kernel is more appropriate to get the best spatial resolution (Fig. 2). Conversely, for models with moderate to large size, low contrast objects, such as liver lesions, a smooth kernel is more appropriate to control image noise (Fig. 2).

**Fig. 3:**
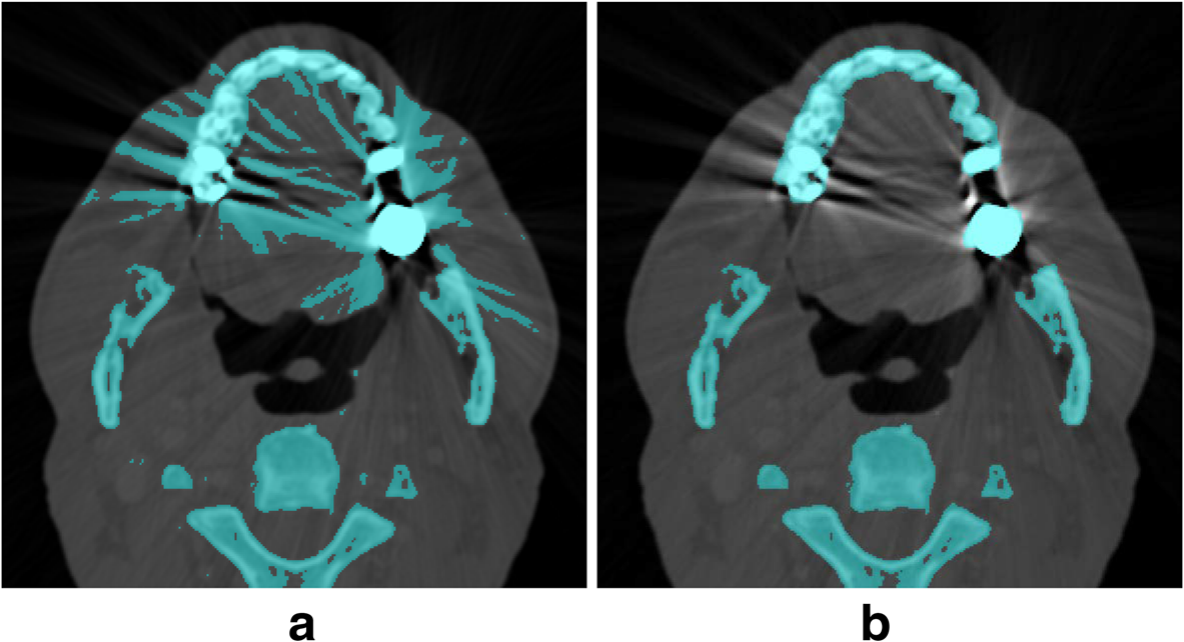
CT images of a patient with dental fillings (**a**) and that after application of a metal artifact reduction algorithm (**b**). Pixels in blue color were results of thresholding segmentation, aiming to separate bone from soft tissues. Metal artifacts contaminated the segmentation in the original images (**a**) while was removed in the image with metal artifact reduction algorithm (**b**)

Novel imaging techniques should be adopted to assist the process of 3D printing to improve accuracy and efficiency. For example, both bone and iodine-enhanced vessels have high CT numbers and show up as bright structures in regular CT images. It can be challenging to separate such bones and vessels. Dual-energy CT, which uses the energy dependence of the x-ray attenuation coefficient, can easily differentiate these two materials [25]. In this case, a ‘bone removal’ process can be performed to remove bones while leaving iodine enhanced vessels. Thus, dual-energy CT acquisitions may be desirable for vascular models. Another challenge frequently encountered in 3D printing is image distortion caused from artifacts in patients who have metal implants. Metal artifacts substantially degrade the image quality of both CT and MRI, and contaminate surrounding anatomy. Novel metal artifact reduction techniques can be used to reduce metal artifacts, consequently improve the efficiency and accuracy of 3D printing (Fig. 3) [26].

### Segmentation and processing

Segmentation and processing are critical steps in transforming medical images into physical models. The goal is to separate the organ of interest from the surrounding anatomy and prepare STL files that are ready for the printer. It is important to understand the number of ways these steps can impact the accuracy of the final 3D printed models.

The first step is to convert DICOM (Digital Imaging and Communications in Medicine) images, standard data format for storing and transmitting information in medical imaging, into segmentation software. There are many segmentation software tools; some proprietary and others freeware. At our institute, Mimics/3matic (Materialise, Belgium) is used for segmentation. Mimics/3Matics has an FDA 510 K clearance for its defined intended use. There are both automatic and manual tools for segmentation. These include, but are not limited to, thresholding based on density signal magnitude, region growing based on continuity of selected signal, and addition, subtraction and filling tools. All staff should be well trained in the optimal use of the segmentation software tools available in their lab.

Automatic segmentation tools cannot be totally relied upon to impart the advanced medical knowledge of anatomy and pathology that is essential for high quality medical segmentation. Depending on the complexity of the model there can be a number of varied steps in the segmentation process, including hand segmentation, which can be time intensive. All segmentation involves some level of judgement on what to include, how to include it and what not to include. Early discussion with the surgeon is essential in order to understand what is important to include in the model. Experienced technologists and radiologists with knowledge of anatomy, pathology and medical imaging techniques are critical for quality segmentation. Radiologists are the most skilled and trained personnel in this area and should oversee the segmentation process.

**Fig. 4:**
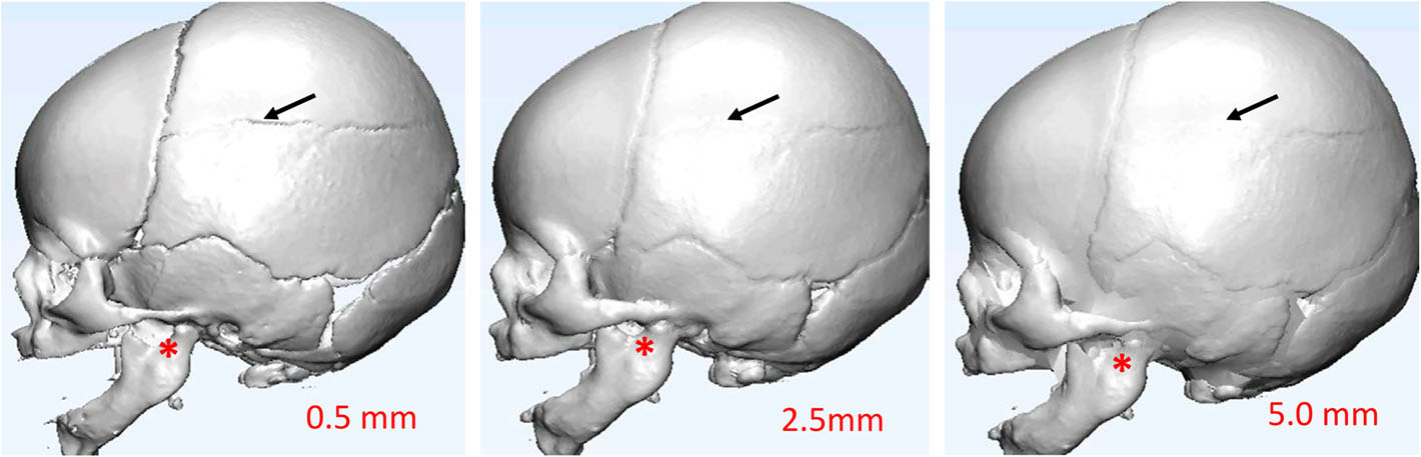
Infant skull wrapping with different parameters. Excessive wrapping shows loss of detail in the cranial sutures (*black arrow*) including normal variant accessory parietal suture, and loss of detail and inaccurate representation of anatomic size in the nose, zygoma, cranial vault, and mandible (*red asterisk*)

**Fig. 5:**
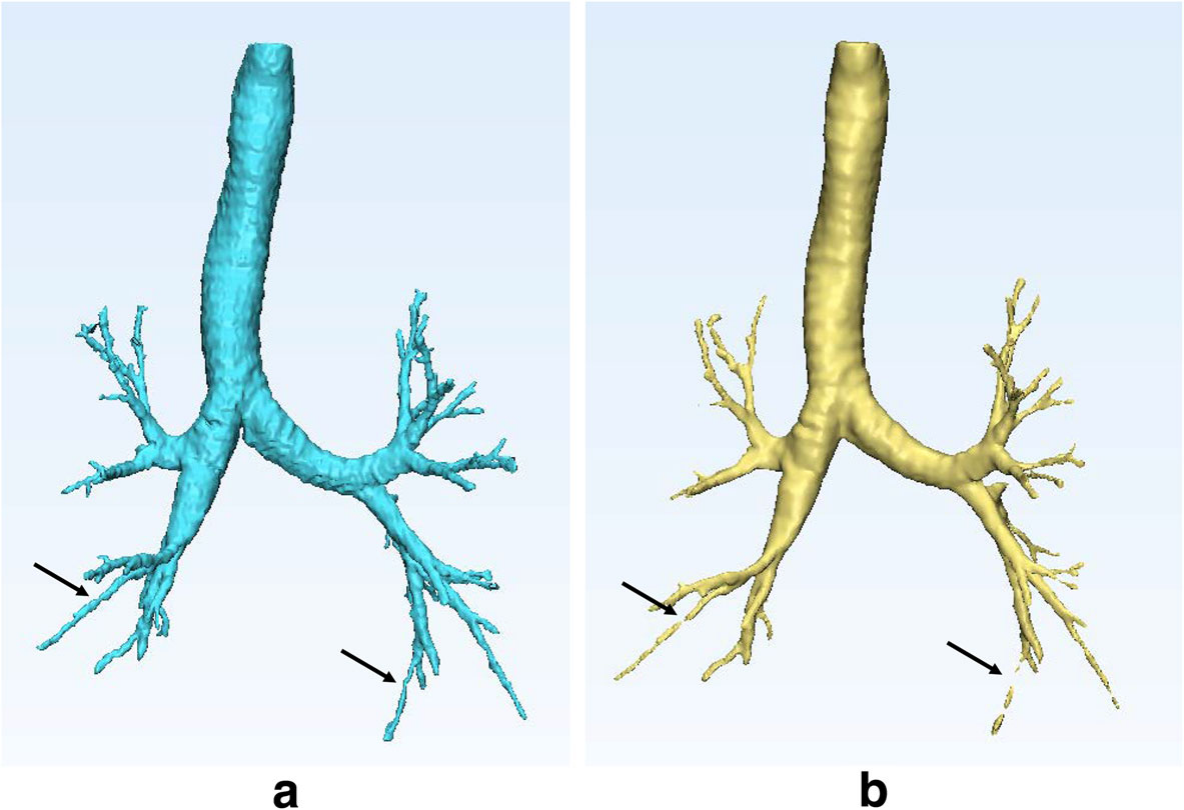
A trachea model without (**a**) and with (**b**) wrapping process. The wrapping process removes distal branches of the trachea (*arrows*)

After segmentation or separation of the critical structures, the data are converted into STL files, the standard file format for 3D printing. The STL mesh files, made up of triangles of various shapes and sizes, are processed to varying degrees in order to be accepted by the 3D printing computer. The computer reconstructs or “slices” the STLs files into thin horizontal layers and prints them. There are many processes for fixing or optimizing the mesh for printing including optimizing the shape and number of triangles and decreasing inverted triangles and bad edges. The software also includes methods to fill holes, connect, smooth and expand the mesh surfaces. These processes are used to “clean up” the mesh to allow for printing and to improve the appearance to more closely reflect the source data. However, these processes have limitations and can inadvertently change the appearance of the model, for examples, removing fine structures from the original model and over smoothing and wrapping surfaces so they no longer reflect the source data (Figs. 4 and 5).

**Fig. 6:**
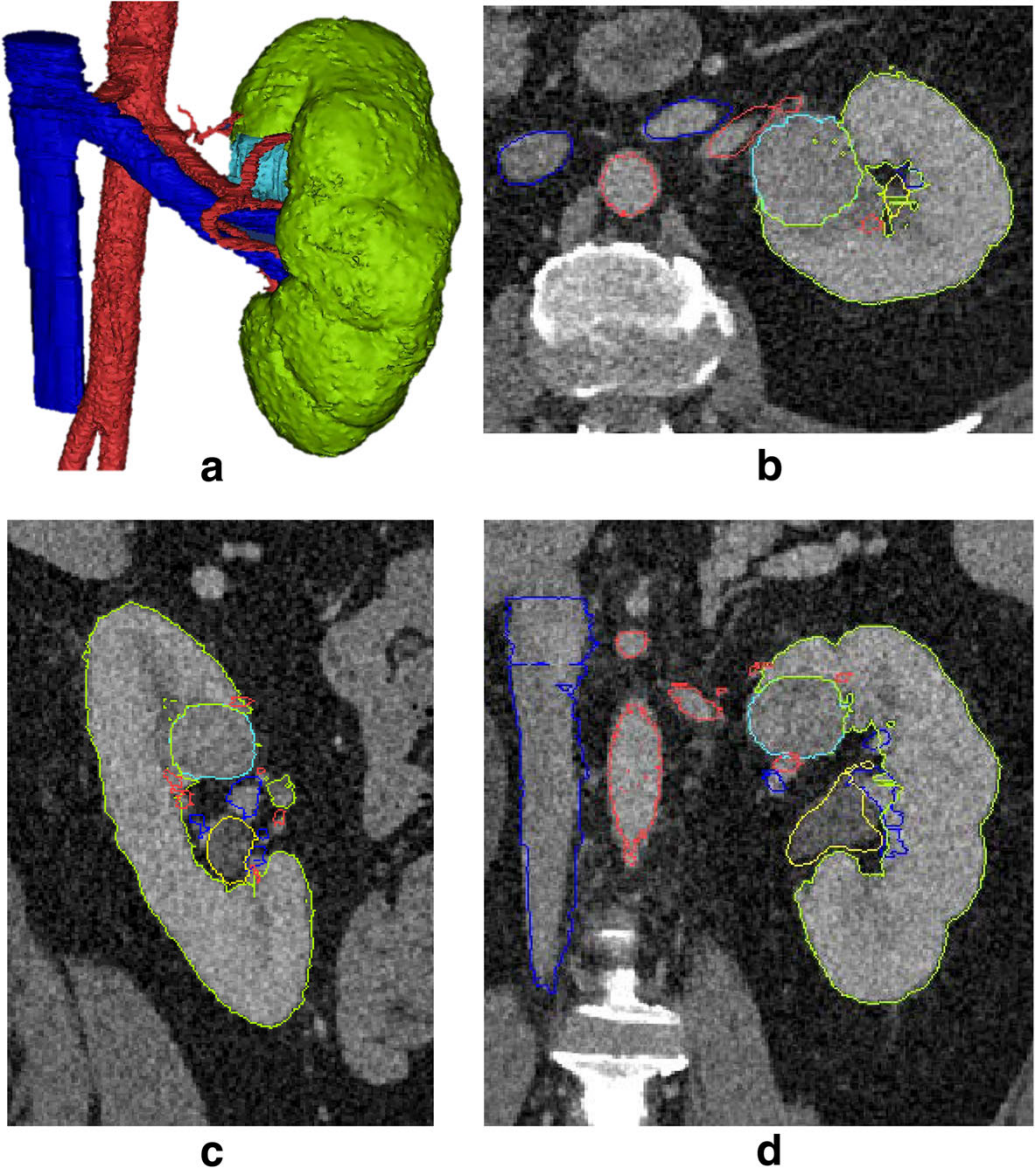
A renal mass model (**a**) with segmented results overlaid on original images in axial (**b**), sagittal (**c**) and coronal (**d**) planes. Different *colors* indicate different segmented objects

It is essential to check for accuracy of the segmentation and processing as the last step before printing. This can be achieved by overlaying the final STL files on top of the original source images (Fig. 6). Users should inspect the entire image set in all three (axial, coronal and sagittal) planes as errors may be more obvious in one plane than the other. This is especially important if the segmentation process is performed primarily in one plane (e.g. axial). Final approval from the radiologist should be obtained before sending the model to the printer.

### QA of printing process

**Fig. 7:**
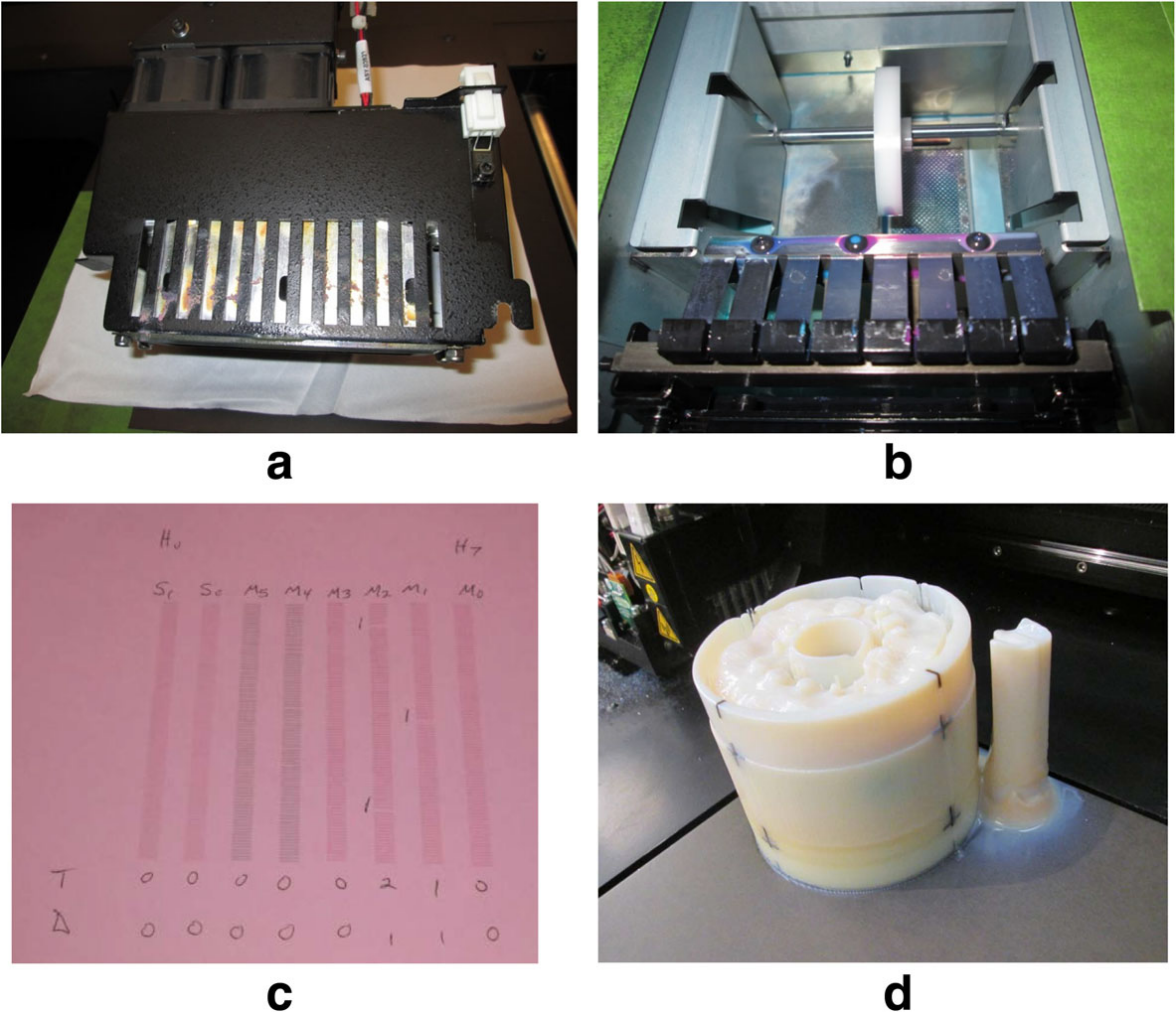
Printer head (**a**) and wiper (**b**) that need cleaning after each printing job. A test pattern run to ensure the heads are not getting clogged (**c**). Under-calibrated UV lamps result in unsolidified parts (**d**)

When a system is being used as a medical device it is subject to more stringent requirements in terms of reliability, accuracy and reproducibility than non-medical devices and systems. As such, additional attention should be paid to the periodic maintenance and testing of medical 3D printers. It is strongly recommended that users follow all specific instructions from the manufacturer regarding routine maintenance with the additional caveat that increased testing frequency or more stringent tests may need to be developed to minimize equipment failure and downtime. The PolyJet printer used at our institution (Objet 500, Stratasys, Eden Prarie, MN) lays down a thin layer of liquid polymer which is immediately cured by a UV light. Its printer head and wiper are cleaned after each printing job and a test pattern run to ensure the heads are not getting clogged (Fig. 7a–c). UV lamp calibration is also performed periodically, which is critical, as under-calibrated UV lamps result in unsolidified parts (Fig. 7d), while over-calibrated UV lamps results in solidified printing material inside the head and pipeline that could clog the print head and interfere with laying down the liquid material.

After the model has been printed, model cleaning is performed to remove supporting materials. Care should be taken that all residual materials have been removed, without removing any structures of the model, especially delicate ones. A visual check should be performed to confirm that the model is as it was intended.

In addition to the regular maintenance performed after each print job, weekly and monthly maintenance is performed by the on-site supporting staff and preventive maintenance is performed by a technician from the manufacturer after every 3500 print hours. This roughly translates to a yearly preventive maintenance appointment. Maintenance includes the replacement of worn plates and tubing, as well as several in-depth factory level calibrations and tests to ensure the printer functions optimally. Close working relationships with 3D printing and software companies are important to ensure optimal maintenance and use of the printer and software, especially as medical institutes initially gain experience and develop expertise.

### Validation of printed model

After construction, physical measurements of the anatomic model should be performed. For example, the distance between landmarks can be measured with a caliper, and angles can be measured with a protractor. However, these measurements are usually limited in their ability to validate the completed model.

Another approach is to use imaging techniques to assist in the validation of the models [27, 28]. The printed model can be scanned using a high resolution imaging modality, e.g. a CT scanner or 3D laser scanner; the imaging modality must provide high resolution and fidelity to avoid compounding errors. In this study, we used an ultra-high resolution mode of a commercial CT system having an resolution of 0.2 mm in plane and 0.4 mm off plane [29–31]. A high radiation dose was used to eliminate the influence of image noise. Images of the scanned model were imported into the Mimics software. Since the model was scanned in air using a high dose, a simple threshold was sufficient to segment out the model and build the virtual model. The virtual model of the scanned printed model was registered to the virtual model segmented from original patient images. After registration, the agreement/disagreement of these two virtual models was analyzed (Analyze module in 3-matic, Materialise, Belgium). The distance between the virtual model segmented from original patient images and the virtual model built from CT images of the scanned printed model was calculated on a point-by-point basis. Statistical information regarding the distance distribution, such as min, max, mean and standard deviation was also obtained.

### Phantom-based QA process

Phantoms are commonly used during QA in medical imaging. Similarly, phantoms provide unique benefits during the QA process of 3D printing. Specifically, a phantom can be used to test the entire 3D printing process, including the major steps of imaging, segmentation, and printing. The size and geometry of the phantom is precisely defined and thereby serves as a gold standard for quantitative comparisons, based on which benchmarks can be obtained and operational limits can be set. Finally, the phantom-based QA process is repeatedly performed at set intervals to obtain longitudinal data regarding the stability of the printer calibrations and performance. During the course of the development of our in-house QA program, two generations of phantoms have been developed. Other phantoms were also reported in the literature used for additive manufacturing of metals for biomedical applications [32].

### First generation QA phantom

Our first generation phantom is an imaging phantom originally used to test resolution in imaging system. The phantom consists of a plastic block containing 11 groups of line-pair patterns. For each group, there are 5 air openings, each with the same size as the adjacent bar. For different groups of line pair patterns, the size of the opening gradually becomes smaller and smaller. The overall size of the phantom and the air openings is known. This phantom can be used to test geometric accuracy and resolution.

Using this phantom, we developed a QA procedure that involved processing the phantom using the same procedure as that of a patient model. It was first imaged on a CT scanner and DICOM images were reconstructed. These images were loaded into the Mimics software (Materialise, Belgium) and a threshold-based segmentation was performed. STL files were generated and exported to the 3D printer for printing. The printed model was cleaned following the same procedure as that of patient models. After the QA model had been printed, the size of the air opening of each group of line pairs was measured using a caliper. These measurements were then compared to the known size of the phantom and the difference was calculated. Influence of imaging technique (such as reconstruction kernel) and processing technique (such as wrapping) can be demonstrated using this QA phantom.

### Rationale and development of second generation QA phantom

We have developed a second generation phantom that includes additional test objects and shapes and has been designed to more comprehensively test all facets of the 3D printing process. It was designed to have a relatively small size so it can be printed relatively quickly as a stand-alone print process or added to an existing printer build tray. The phantom was designed to include both geometric objects and anatomic contours, and aims to test multiple aspects related to 3D printing, such as geometric accuracy, spatial resolution, curvature, and shape fidelity. Practical considerations were taken into account during the phantom design, such as using measurable objects, using small dimensions to minimize the amount of materials and printing time, and the ability to remove supporting materials. The phantom was designed in 3-matic (Materialise, Belgium).

## Results

### Validation of printed model

**Fig. 8:**
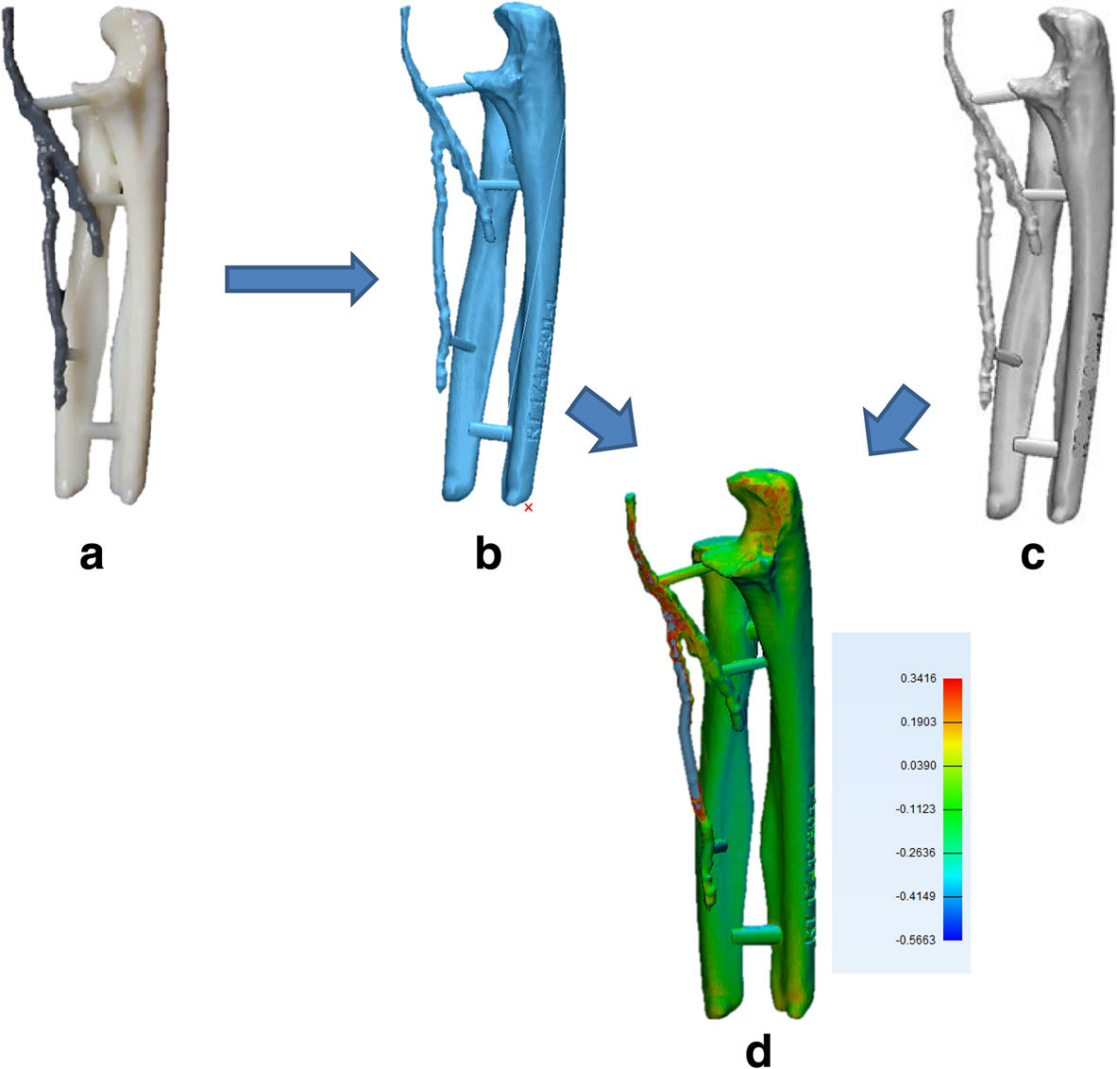
The printed 3D model (**a**) was imaged using a high resolution CT scan from which a virtual model was constructed (**b**). This virtual model was registered to the one derived from original patient CT images (**c**). The distance between these two models was calculated after registration and *color coded* (**d**) to show the agreement at each location of the model

Figure 8 showed a 3D printed radial-ulna model. This printed model was imaged using a high resolution CT scan and a virtual model was derived from the CT images of the printed model and registered to the original virtual model derived from patient CT images. The distance between these two was calculated after registration and color coded, from which points with high deviation were easily identified. The distance ranged from −0.57 to 0.34 mm for this model, with a mean of −0.12 mm and a standard deviation of 0.17 mm.

**Fig. 9:**
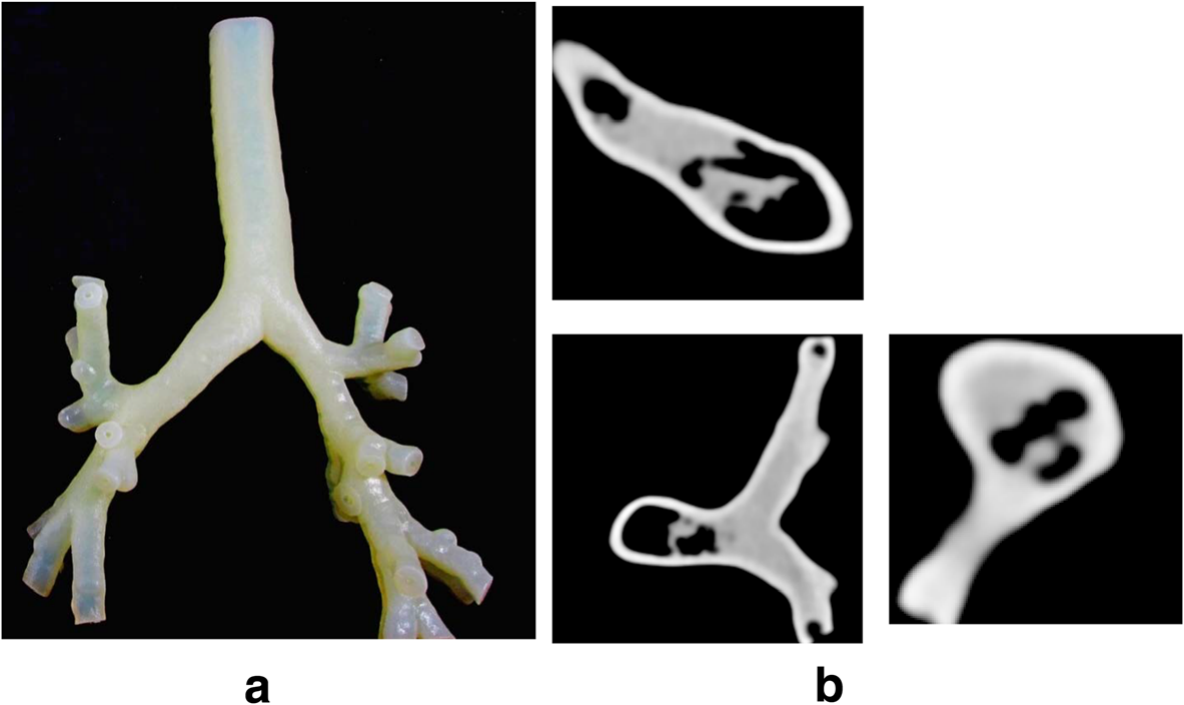
CT scan of a trachea model (**a**) showed residual supporting materials inside (**b**)

**Fig. 10:**
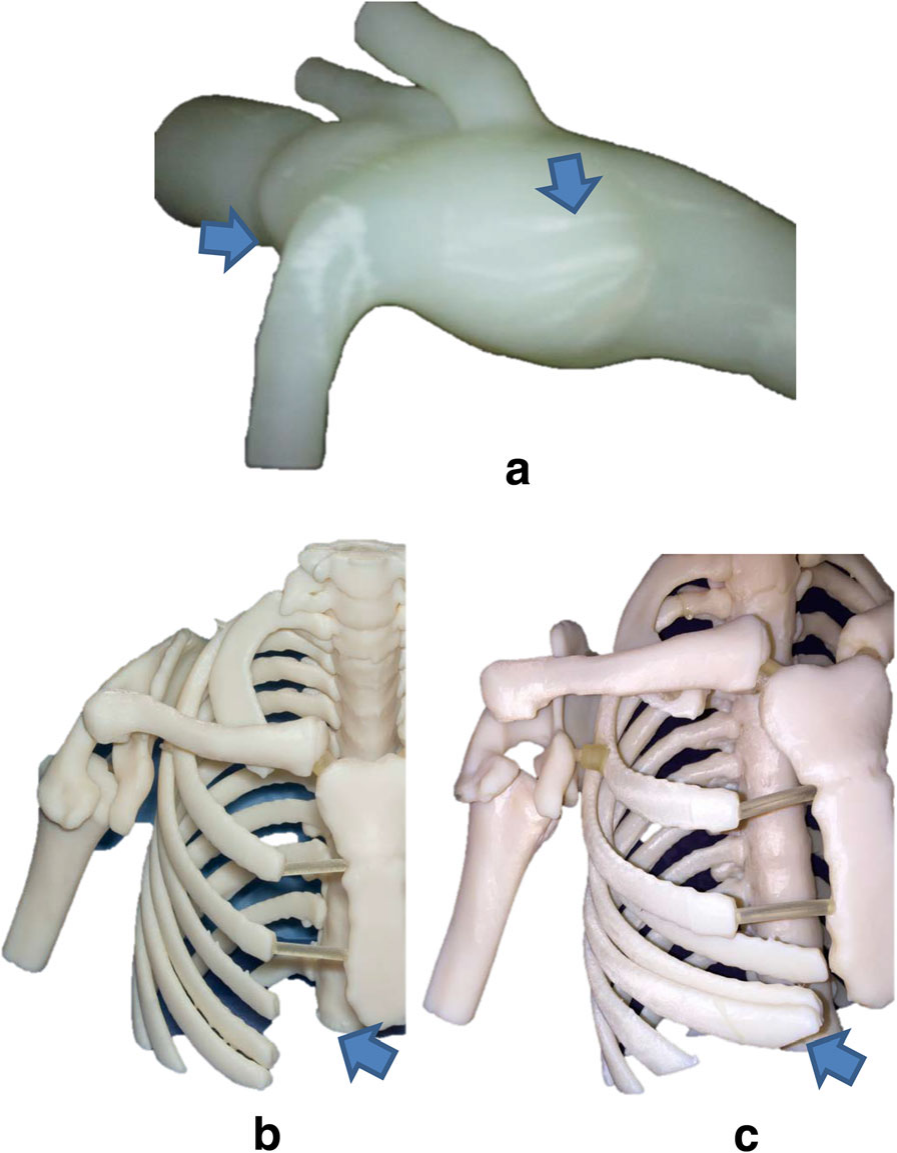
An aorta model showed cracks (**a**) and a rib cage model (**b**) showed deformity after placing on shelf longer than 1 year (**c**)

Special care should be taken for models involving hollow spaces, such as vascular models. If the hollow space is important, it should be checked for residual supporting materials. A CT scan can easily reveal the lumen of the vessel and whether there are residual supporting materials (Fig. 9). Material integrity should also be checked if models are used clinically over a long time period. Figure 10 shows cracks on an aorta model and deformation of ribs on a chest model after being left on a shelf for over a year. Recheck of model integrity should be performed if models are to be stored for an extended time period after they are built. Longitudinal scans of the model over time can determine how long the integrity of the model is maintained for scenarios where models are needed for a length of time after they are printed.

### First generation QA phantom

**Fig. 11:**
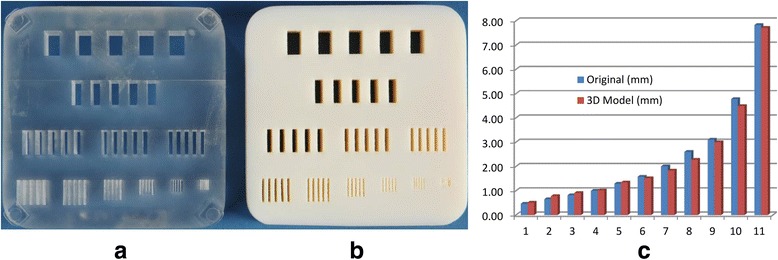
A picture of the 1^st^ generation QA phantom (**a**) with 11 groups of line patterns, and the 3D printed model (**b**). Measurements of the air gap between printed bars from the original and 3D model of the QA phantom showed how accurate the 3D printed model represented the original phantom (**c**)

Figure 11 shows photos of the first generation QA phantom including the original phantom (Fig. 11a) and the 3D printed phantom (Fig. 11b). The size of the air openings for each group was measured for both the original and 3D printed phantoms. The difference of the air gaps between the original and 3D printed QA phantom was plotted for each of the 11 groups (Fig. 11c), and was found to be between −0.32 and +0.13 mm.

**Fig. 12:**
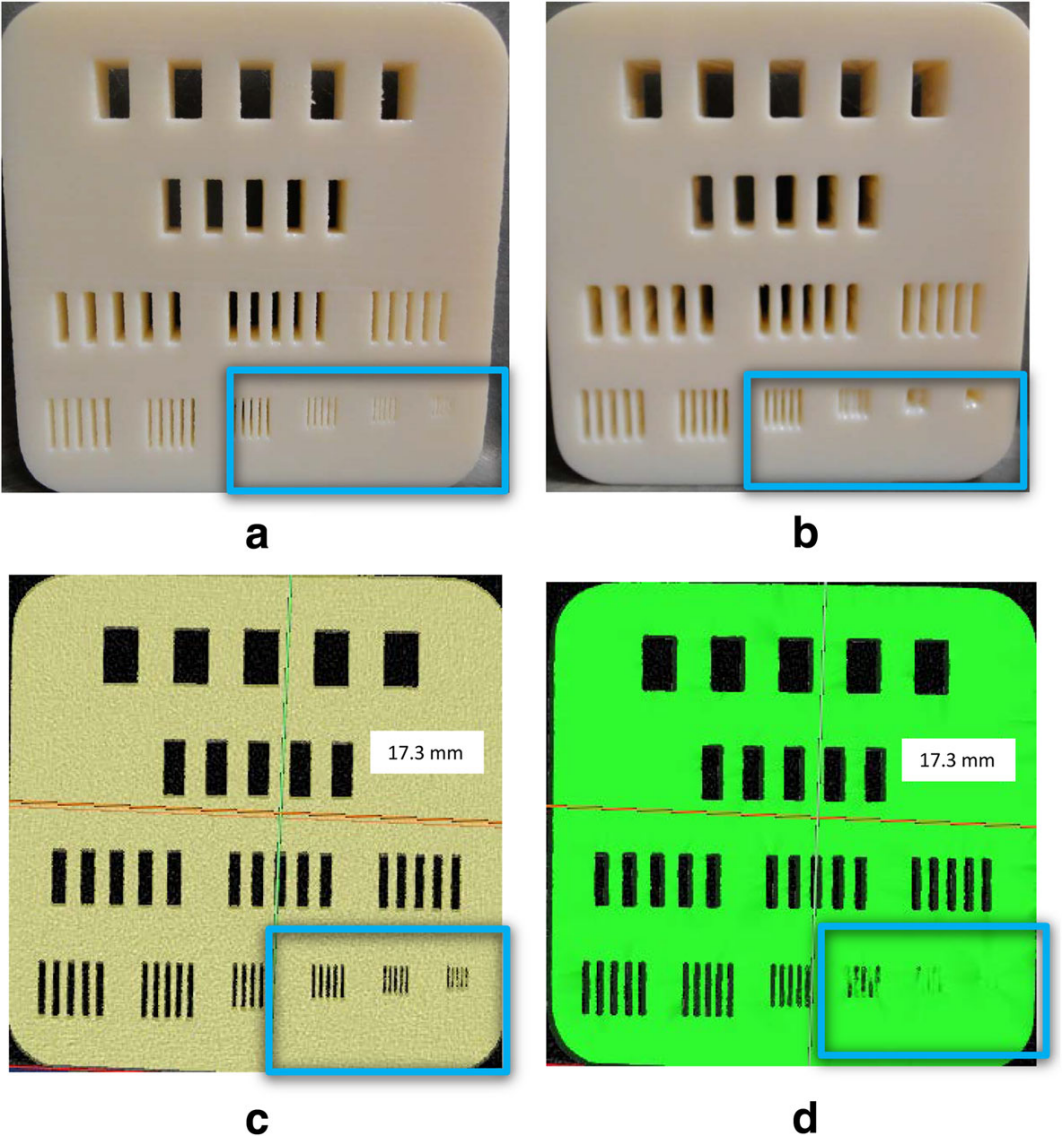
The impact of imaging techniques and processing methods can be tested with the QA phantom. The same QA phantom scanned with bone imaging technique and reconstructed with a sharp kernel (**a**) and body imaging technique with a soft kernel (**b**) showed differences in the *smallest bar* patterns that could be differentiated. Similar observation for models with the same imaging data but processed without (**c**) and with (**d**) wrapping

Figure 12 shows the impact of imaging techniques and processing methods tested with the QA phantom. The same QA phantom scanned with bone imaging technique and reconstructed with sharp kernel (Fig. 12a) showed more differentiable bar patterns compared to that scanned with body imaging technique and reconstructed with soft kernel (Fig. 12b). Similar observation for models with the same imaging data but processed without (Fig. 12c) and with (Fig. 12d) wrapping.

### Second generation QA phantom

**Fig. 13:**
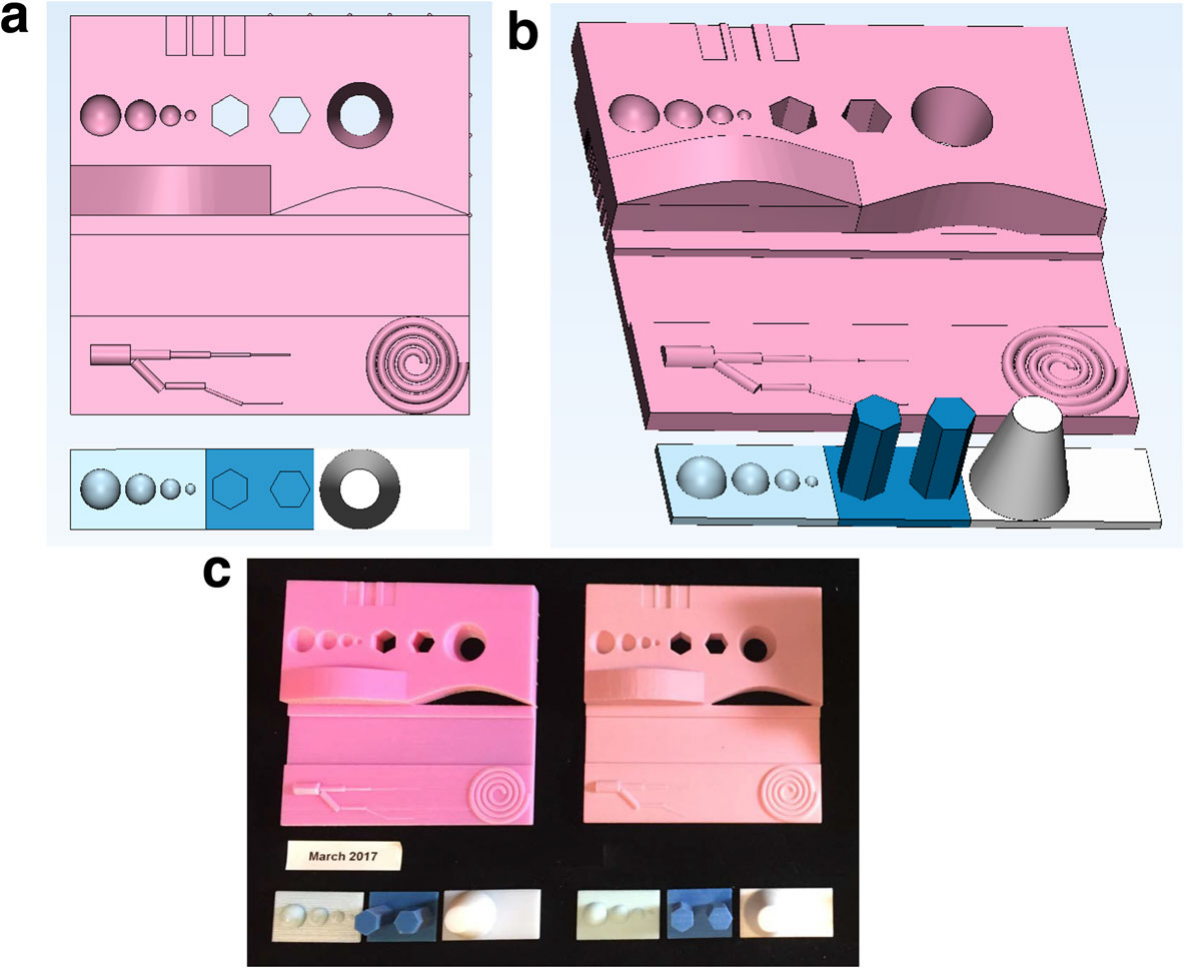
Schematic of the final design of the 2^nd^ generation QA phantom from different views (**a**, **b**), and photos of the printed QA phantom on the Stratasys Objet500 Connex3 (*left*) and 3D Systems ProJet 660Pro (*right*) (**c**)

Figure 13 shows the 3D rendered version of the phantom and identifies the individual test objects. Table 1 provides dimensions of each test object, the specific parameter being evaluated and its dimensions. The phantom includes both geometric objects and anatomic contours, and includes both positive (standing out) and negative (embedded in) objects. Of note, the positive base-the side of the phantom containing positive test objects -contains three test objects (spheres, hexagons, and cone) that have been printed on separate bases that fit into the positive base. These objects are positives of the shapes on the negative base and have been designed to fit into their negative counter parts. This enables a rapid, qualitative assessment of the quality of the print job by placing the positive objects in the negative forms. The spiral and cylinder patterns on the positive base have been designed to replicate specific anatomic shapes that are challenging to reproduce on a 3D printer, specifically the spiral pattern found within the cochlea of the ear and the small diameter branching structures found in the peripheral vascular tree of the human body.

**Table 1:** Second generation anatomic 3D modeling QA phantom. Specific test objects and their dimensions are described and are also illustrated in Fig. 13

Test object	Test	Dimensions
Line pair pattern	Spatial resolution	Line pairs(lp) at 0.5, 1.0, and 2.0 lp/mm along each physical axis
Concave/Convex surface	Curvilinear surface accuracy	Height-7.5 mm Length-50 mm Width-12.5 mm Depth-10.0 mm
Spheres	Shape fidelity	Semi spheres at diameters of 2.5 mm, 5.0 mm, and 7.5 mm
Cones	Shape fidelity	Positive cone: Bottom diameter-20 mm Top diameter-10 mm Height-20 mm Negative cone: Top diameter-16.5 mm Bottom diameter-10 mm Height-7.5 mm
Hexagonal Cylinder	Rectilinear geometric accuracy	Width-10 mm Height-20 mm
Positive base	Volumetric accuracy & temporal stability	Width-50 mm Length-100 mm Height-5 mm
Negative base	Volumetric accuracy & temporal stability	Width-50 mm Length-100 mm Height-12.5 mm
Ruler	Spatial accuracy	Five markers placed at 10 mm spacing along physical X and Y edges of phantom on negative base.
Spiral	Anatomic	Inner diameter-1.8 mm Outer diameter-2.5 mm Spiral start-end separation-10 mm
Vascular tree	Anatomic	Cylinders of uniform length of 10 mm with diameters of 5 mm, 2.5 mm, 1.25 mm, 0.75 mm and 0.5 mm. Two outer branches at diameters of 2.5 mm and 0.75 mm placed at 45° and 25° respectively to the inner cylinders.

Errors introduced at the time of printing can be easily assessed by the accuracy of the ‘fit’ of the positive object into the complimentary negative form. Long term stability of the phantom can be assessed by either serial temporal measurements or by comparing measurements taken at the time of printing at some predetermined interval. Additional physical measurements of the dimensions of the object can be obtained at any time by high resolution CT imaging, 3D surface scanning or physical measurement of the phantom’s dimensions using precision mechanical calipers.

Both first and second generation phantoms provide the ability to evaluate material stability in response to environmental factors such as temperature, pressure and humidity, as well as the temporal stability of the materials used to create these models. Thus, it is recommended to print either model, or a variant thereof, at a fixed frequency (monthly or quarterly) and to perform routine testing of the phantom by either physically measuring the object or by more sophisticated methods involving imaging and computer analysis. Data from these measurements should be recorded and analyzed. Such analysis provides valuable information regarding the long-term stability of the printed materials, identifies factors that impact the accuracy of the models, and also provides valuable information on whether or not the 3D printer is in failure. The QA process should be standardized and an operation manual created to document that the QA process is performed precisely and consistently.

### Identification and documentation

Each 3D printed anatomic model is labeled with a unique patient identifier for traceability. This could be achieved either directly using patient information (e.g. last name or clinic number) or generating a specific unique identifier that can be linked to the patient information. Any patient information should be protected according to the Health Insurance Portability and Accountability Act (HIPAA), similar to other areas of medical practice. In certain scenarios, additional identifiers, such as left or right side or mirror image anatomy, may be necessary. These labels can be imprinted on anatomic models through a computer aided design program.

For long-term record keeping, the segmentation, STL files, and photographs of each model are stored in a designated repository. In addition, a photograph of each model is placed in the patient’s medical record. The STL files can be used to reprint the model at any time, without the need to re-segment patient images and redesign the parts. All files should be saved on secured large capacity storage servers with daily backup.

## Discussion

The past decade has seen remarkable growth in the use of 3D printing in medicine. The growth has been fueled by the development of high resolution imaging studies merging with the rapid development of 3D printing technologies, and the development of new printing materials. These advances have resulted in reductions in the costs associated with creating high resolution medical models. The evolution of this disruptive technology is expected to revolutionize medical practice.

While much attention has been focused on the application of 3D printing in medicine, less attention has been given to ensuring that the 3D printed model is a true and accurate representation of the physical object under study. If this technology is to be widely integrated into advanced medical practices, ensuring the physical accuracy and reproducibility of 3D models by means of a comprehensive QA program is essential.

This work addresses these concerns by providing the necessary framework for developing and maintaining a QA program for 3D printing. Incorporation of existing imaging quality procedures forms the foundation for the program. It begins with acquisition of high quality imaging data using ACR accredited personnel and machines. Accuracy of segmentation by trained staff and awareness of the impact of processing STL files is the second step in the program. Verification and validation of the 3D printing process including phantom testing and model analysis is the third step. Unique identification of models along with documentation in patient medical record is also an important part of the process. The QA program delineated in this work thus describes an end-to-end testing of the entire 3D printing process.

Among the three major steps in 3D printing: imaging, segmentation, and printing; the segmentation step is the most challenging with regards to QA. Significant effort was reported investigating accuracy when printing boney anatomy, with scanning bone in air, water and in situ with subsequent removal of soft tissues to reveal bone dimensions to perform the comparison [33–36]. The accuracy of segmenting highly depends on the image quality, such as spatial resolution and contrast to noise ratio. For models scanned in air, such as the QA phantom or the printed model, the segmentation is relatively easy given the high contrast between the model and the air background. It becomes much more challenging for patient cases, especially for segmentation of different types of soft tissues with low contrast. Therefore, it is essential to check for accuracy of the segmentation before printing. We recommend checking the overlaps between final STL files and the original source images. Medical knowledge about anatomy and pathology is required to judge the accuracy of segmentation. Work presented here represents early and simple approaches. Further development on objective, quantitative and time-effective QA method in segmentation is desired.

Phantoms are critical for the QA process. Two generations of QA phantoms have been developed and used in this study. A similar QA process and testing phantom like the first generation QA phantom can be easily developed by those starting a 3D medical modeling program. In this way, experience and expertise can be gained without creating too complex of a QA program which may absorb limited resources and personnel. However, the 2^nd^ generation phantom has more test objects and shapes which can more comprehensively test all facets of the 3D printing process.

One of the validation methods presented in this study was to scan a printed model and compare with the initial segmentation. This method works well to evaluate the total shape of the printed model, but challenges exist to evaluate internal structures of models with multiple components. For models with multiple components, materials with different HU values should be carefully selected to represent different components. The HU range of available printing materials could be a limiting factor in this approach [14]. The alignment of the source STL and that resulting from scanning the printed model could be potentially difficult in certain scenarios. Although automatic alignment tools exist in some software, the accuracy of the alignment should be carefully evaluated and manual adjustment may be needed. Misalignment will result in errors in the evaluation of agreement. Also, appropriate imaging modality and imaging protocols should be used. High resolution, high geometric accuracy, low noise, and artifact-free imaging method should be used to avoid additional errors introduced by the imaging process in alignment and measurement. This method also has challenges for models built with flexible materials. Since these models may deform after being printed, it is not an easy task to ensure that the model maintains its original shape during the scan. For this scenario, it is helpful to build some supporting structures while building the model to support the model in order to have it retain the original shape it has in the patient. This way, the same method can be used. Given these potential limitations, this method should be carefully evaluated for the specific applications before used as a routine QA process.

There are several limitations to this work. First, the protocols are based on experience with a single type of 3D printer and with segmentation software from a single vendor. The general framework and concepts of this QA program, though, can be extended to other types of printers with appropriate adjustments made according to the specific printing technology and to type of segmentation software. Secondly, our experience relies heavily on the use of CT imaging data which is used for the majority of our models as CT provide high spatial resolution and high geometric accuracy, both of which are critical for 3D printed models used in medicine. However, general principles outlined in this paper apply to 3D printing using other imaging modalities too. MRI data is increasing used as an adjunct to the CT data as higher resolution MRI imaging sequences are being developed. The use of 3D Ultrasound data is still in early stages of exploration for 3D printing. Finally, the QA program does not provide specific and quantifiable standard for 3D printing. As this technology evolves substantial QA data from multiple institutions needs to be accumulated over time so that appropriate specific and quantifiable QA standard will be developed and adopted by the medical 3D printing community.

## Conclusions

In conclusion, this work describes the development of a comprehensive QA program for 3D printing in medicine. It is the hope that the methodologies here described will contribute toward the growing body of work needed to establish standards for QA programs for medical 3D printing.
